# A Review of Toxicological Profile of Fentanyl—A 2024 Update

**DOI:** 10.3390/toxics12100690

**Published:** 2024-09-24

**Authors:** Jessica Williamson, Ali Kermanizadeh

**Affiliations:** College of Science and Engineering, University of Derby, Derby DE22 1GB, UK

**Keywords:** fentanyl, human health, toxicity, demography, post-mortem quantification

## Abstract

Fentanyl and its analogues are synthetic opioids of varying potencies that are unfortunately heavily abused. Over the last 15 years, fentanyl and its analogues have contributed to the increasing prominence of hospitalisation and numerous deaths due to drug overdose. In this comprehensive literature review, the mechanism of toxicity of the drug in humans is evaluated. A systematic approach was used whereby the relevant literature has been detailed where the toxicity of fentanyl and/or its analogues to different organs/systems were investigated. Furthermore, the review covers the post-mortem toxicological data and demographic information from past fatal cases where fentanyl was believed to be involved. Such insight into fentanyl toxicity is useful as an aid to better understand the toxic doses of the drug and the suspected mechanism of action and the unexpected complications associated with overdose incidences involving the drug. Finally, the review offers an overview of the traditional and emerging test systems used to investigate the adverse effects of fentanyl on human health.

## 1. Introduction

Fentanyl and its analogues are classed as synthetic opioids (similar to heroin and morphine), meaning that they are full agonists of differentiating potencies at the μ-opioid receptor [1]. Fentanyl molecules are related to that of phenoperidine, which was originally synthesized by adding and/or replacing numerous chemical entities to the meperidine molecule, except fentanyl is more than 10 times more potent [2]. Currently, two types of fentanyl are available, pharmaceutical fentanyl and non-pharmaceutical fentanyl, more commonly referred to as illicitly manufactured fentanyl (IMF). Pharmaceutical fentanyl is the drug that is a medically prescribed formulation commonly used in the management of pain, available as tablets, sprays, lozenges, skin patches, or injections. Non-pharmaceutical fentanyl (or IMF) is the illegally produced form of the drug and is often distributed as counterfeit prescription pills or combined with other illicit drugs like cocaine, heroin, or methamphetamine in a powder or liquid [3]. Fentanyl derivatives belong to a class of novel synthetic opioids that have emerged in an ever-growing illegal drug market of new psychoactive substances [4]. According to the United Nations Office on Drugs and Crime (UNODC), opioids are substances which are central nervous system depressants. They have structural features that allow binding to specific opioid receptors, resulting in morphine-like effects, i.e., analgesia [5].

Fentanyl was first introduced in 1960 as an analgesic, a medicine for pain management, and was approved by the FDA in 1972 for use as an intravenous anaesthetic under the trade name Sublimaze^®^ [1]. Since then, the fentanyl patch has been widely used for the management of chronic pain [2]. Historically, the development of fentanyl as an analgesic agent was founded upon the knowledge that the lipophilicity of a molecule plays an important role in their onset time and potency level, creating the desire to develop a more lipid-soluble opioid to heighten these factors, compared to those previously available [6]. The rationale that the development of a highly potent drug with a greater receptor specificity would have a better safety profile than that of morphine also influenced the synthesis of fentanyl [7].

Over the last two decades, many other medications with a similar chemical structure to that of fentanyl, more commonly known as fentanyl analogues, have been synthesised [7]. Some of these analogues are registered for human use—alfentanil and sufentanil—while others are commonly utilised in veterinary—carfentanil and thiofentanil. Furthermore, other analogues, such as acetylfentanyl and furanylfentanyl, have not been registered for medical use and, therefore, are IMFs [8]. 

In 2024, fentanyl is one of the most frequently used opioids in the medicine availability intravenously, transdermally, and transmucosally [2]. The high lipophilicity and low ionisation of fentanyl molecules favours its absorption across biological membranes within the body, making it a highly effective drug [6]. The drug’s fast onset rate contributes to its growing popularity, standing at rates of analgesia of as little as 1 to 2 min after intravenous administration, 5 to 10 min in sublingual (mouth) and intranasal (nose) sprays, or 10 to 15 min after most types of transmucosal administration such as lozenges, tablets, and films [2]. Fentanyl has been found to be the superior choice compared to other opioids given its higher potency, around 50 to 100 times more than that of morphine, shorter onset time, and duration of action, along with other beneficial properties such as the faster recovery time after extubating from anaesthesia and its manufacturing process using readily available precursors, resulting in the cheaper production of the drug [9]. 

Like other μ-opioid receptor agonists, fentanyl produces the typical clinical effects within the body of miosis, depression of the central nervous system (CNS), and respiratory depression [1]. The μ-opioid receptor is a G-protein coupled receptor (GPCR) which is encoded by specific structural genes, which, in turn, encodes for the seven-transmembrane feature that all GPCRs possess. G-proteins themselves consist of an α-subunit, a y-subunit, and a β-subunit, which are significant in the signalling pathway [10]. When an agonist like fentanyl binds the receptor, a conformational change occurs at the receptor which results in the dissociation of the G-protein subunits from the receptor, and, in turn, the subunits’ subsequent activities result in decreased calcium influx, decreased cyclic adenosine monophosphate (cAMP) production, and increased potassium efflux. Thus, the neuronal excitability and, therefore, signal transmission are decreased, which is responsible for the common analgesic effects of such opioids [11]. μ-opioid receptors are generally located in the brain, spinal cord, and peripheral nociceptors. They are present within the ascending pain transmission system located at the pre- and postsynaptic sites in the dorsal horn of the spinal cord, the cortex, thalamus, and the brain stem. They are also located in the periaqueductal gray, the nucleus raphe magnus, and the rostral ventral medulla, which are regions of significance in the descending pain transmission system [12]. Other common side effects associated with the use of fentanyl include drowsiness, relaxation, euphoria, sedation, fatigue, dizziness, anxiety, hallucinations, nausea, vomiting, constipation, retention of urine, apnea from respiratory depression, and lack of consciousness [8]. 

In 2019, approximately 600,000 deaths were attributable to drug use, with 80% of these being opioid-related (480,000) [13]. From this number, around 75,000 deaths are understood to be fentanyl-related [14]. For fentanyl, very much like other drugs, men have higher rates of abuse or dependence [15]. However, women are more susceptible to cravings [16]. The issues with regard to fentanyl are further highlighted by the fact that over 900,000 individuals abusing fentanyl products in 2022 in the USA alone [17], with the number of fentanyl trafficking offenses in same year increasing by 460% compared to 2015 [18].

Although fentanyl has been the breakthrough opioid for clinical use, the drug’s potential for abuse and its relation in the rise of overdose deaths, in particular, in the last decade, has become a significant public health risk. Fentanyl-related overdose deaths began to increase after its synthesis and combination with illicitly sourced drugs since 1979, which predominantly occurred in the United States, affecting younger people [19]. An increase occurred in 2006 in the number of fentanyl-related fatalities [20], and, since then, the opioid related mortality rate has increased continuously, for example, more than doubling the numbers of fentanyl-related fatalities between 2012–2014 [21]. Estimates have suggested that the presence of fentanyl and its analogues contributed to nearly half of the reported opioid-related deaths in the United States in 2016 and that the number of deaths under these circumstances nearly quadrupled between 2015–2017 [22]. Although certain properties of fentanyl have made it the superior analgesic (high potency, fast onset time, and duration of desired effect), the same properties could also be considered to contribute to the drug’s higher risk of overdose and fatality [9]. Regarding the illicit production of fentanyl, the rate at which new products become available, in all reality, contributes to the difficulties in the detection and regulation of these products, resulting in the number of cases of fentanyl misuse being underreported [1]. This further highlights the risk that fentanyl and its analogues impose upon the public, given that these opioids are being manufactured and distributed at a much higher pace than they can be recognised, regulated, and seized. 

Given the continually rising number of fatal cases involving fentanyl and fentanyl analogues, an understanding of the mechanisms in which fentanyl and its analogues can induce toxicity within an individual is crucial. Therefore, in this review, the toxicity of fentanyl and its analogues to the body are evaluated. The mechanism of toxicity is discussed in detail. Importantly, data on the toxicity reported following the use of both pharmaceutical and illicit fentanyl will be considered where available. Lastly, considering fentanyl use resulting in fatality, findings from post-mortem cases, including toxicological data and demographics, whereby fentanyl and/or fentanyl analogue use has been attributed to the deceased’s cause of death will be reported.

## 2. Methods

The databases PubMed and Web of Science were searched to identify articles published which investigated the toxicity of fentanyl and/or its analogues, and the associated effects of such toxicity within the body. In this review, the search terms “fentanyl toxicity” and/or “mechanism of toxicity for fentanyl” were utilised for the last literature search conducted on 10 July 2024 (total of 647 manuscripts were identified in the initial search). From the identified publications, duplicates were excluded, and relevant articles were selected based on their title and abstract content. Relevance was attributed if a publication had a focus on fentanyl toxicity and/or the toxicity of fentanyl analogues opposed to any other opioids reported. A publication had to have included one or more of the following features: detail of how fentanyl and/or fentanyl analogue toxicity presented itself in the form of an effect within an organism with a corresponding dosage value of fentanyl and/or fentanyl analogue that caused this induced effect, detail of how a toxicity-induced effect reported was caused via a proposed mechanism, and/or reported post-mortem toxicological data of fentanyl and/or fentanyl analogues whereby this drug was involved in death. Due to the inevitable limitations and exclusionary nature of any literature search, studies that did not include the search terms in the title or did not provide adequate information in the abstract might have been unintentionally omitted.

## 3. Main Body

### 3.1. Hepatic Toxicity

To investigate fentanyl-induced liver toxicity in 2023, a group of male BALB/c mice (aged 7–8 weeks) were administered an intraperitoneal injection of fentanyl at a concentration of 50 μg/kg on day 1, 3, 5, and 7 and at a concentration of 1 mg/kg on day 9, before being euthanised on day 10, followed by the collection of liver samples. Metabolomic profiling of the exposed liver showed significant changes in the profile produced from the fentanyl group and significant differences between the fentanyl group profile compared with the other tested groups (treatments/reversal and control groups) including methyl leucine, xylitol, lactic acid, glycine, and phosphoric acid. Further analysis highlighted some of the significant pathways involved in the altering of liver metabolites following fentanyl administration, with the lactose degradation, Warburg effect, glutamate metabolism, glucose-alanine cycle, and gluconeogenesis all being affected. A histopathological analysis of the liver showed ‘moderate’ liver injury in the mice exposed to fentanyl, including inflammation in the portal tract and central vein, along with findings of fentanyl-induced changes in CYP3A11 (CYP3A4 in humans) and upregulated interleukin-6 (IL6) secretion [23].

A study conducted by Yadav et al. (2020) [24] considered the potential mechanisms by which fentanyl caused toxicity to different organs, including the liver and kidneys. This study used male Swiss albino mice receiving fentanyl intraperitoneally once a day for a period of 21 days. The fentanyl received was <10 mL/kg the body weight of the mice. After 21 days of receiving fentanyl, the authors demonstrated histopathological changes in the liver, manifested as lymphocyte infiltration and degenerative changes including hepatocyte necrosis and pyknosis. Furthermore, the authors describe tubular degeneration, haemorrhage, necrosis, and sloughing off cuboidal cells in the kidneys of fentanyl-exposed animals. This study also looked at a potential mechanism behind fentanyl dependence whereby it was evidenced that fentanyl caused an increase in protein levels of the c-Fos, glucocorticoid receptor (GR), N-methyl-d-aspartate receptor1 (NMDAR1), and μ-opioid receptor (MOR) in the cerebellum and cortex. These results indicate that fentanyl mechanistically induces neural changes that appear to assist in drug addiction.

Another study considered how fentanyl toxicity may differ in burn patients due to polymorphisms, especially in the cytochrome P450 (CYP) family given their association with drug metabolism. This is another condition by which fentanyl may be administered for analgesic purposes [25]. This study used samples taken from 13 adult participants (aged 18 and above) receiving fentanyl for 20% or more total body surface area (TBSA) burns. Genotype analysis was conducted in the plasma from the patients aiming to identify mutated variants of CYP present in the burn patients. The data identified three burn patients with mutant CYP variants (more specifically CYP3A4 and CYP2D6) from which samples were collected at 0, 15, 30, and 60 min following continuous fentanyl administration at 70 μg/min. The fentanyl concentration in plasma, 15 min after administration, was found to be significantly higher in the patients exhibiting the mutant CYP variants compared to the patients exhibiting CYP with normal activity, indicating a decreased clearance of fentanyl in patients with CYP polymorphisms. This suggests an associated decline in the efficient elimination of fentanyl which could result in the potentially life-threatening accumulation of fentanyl within the body [25]. 

### 3.2. Cardiac Toxicity

Tschirhart and colleagues [26] considered the molecular mechanism of fentanyl-induced cardiac toxicity, in particular, the drug’s influence on the cardiac ionic currents. The study used cardiomyocytes isolated from neonatal rats to investigate fentanyl’s effect on the human ether-à-go-go-related gene (hERG) current, important in maintaining a synchronous heart rhythm via the balanced flow of ions. It was found that fentanyl exposure caused the blockage of the hERG current at concentrations of as low as 0.27 μM. This study also found that fentanyl exposure resulted in a prolonged action potential duration in the cardiomyocytes, which can lead to cardiac arrhythmias or even death, caused by the mechanistic blockage of the hERG current.

### 3.3. Neurotoxicity and Behavioural Effects

Fentanyl-induced neurotoxicity was also investigated in a study using male Wistar rats aiming to determine how fentanyl influences the release rates of the neurotransmitters glutamate and GABA in the anterior hypothalamus. The animals were intravenously exposed to fentanyl at a dose of 10 μg/kg, along with given an intrahypothalamic administration at a concentration of 0.1 nmol/min. Both administration routes exhibited the same outcome, whereby fentanyl exposure significantly decreased the release rate of glutamate while significantly increasing the release rate of GABA. These findings demonstrate that fentanyl can act on the opioid–glutamatergic transmission pathway by altering the release rate of neurotransmitters in the hypothalamus [27].

Next, the effects of chronic fentanyl exposure on the cerebral cortex was assessed in an adult male Balb/c mice model. The animals were intraperitoneally exposed to the drug at doses of 0.05 or 0.1 mg/kg once daily for a period of 5 weeks. The data showed that fentanyl induced apoptosis in the cerebral cortex, mechanistically evidenced via the significantly increased Bax expression and significantly decreased Bcl-2 expression. Furthermore, it was demonstrated that fentanyl-induced inflammation in the cerebral cortex manifested as increased levels of IL-1ß, IL6, and TNF-α, as well as significant oxidative stress. The microscopic examination of the cortex found white matter fragmentation. Finally, fentanyl reduced the expression of NMDA receptors and dopamine receptors whilst elevating epidermal growth factor (EGF) levels, which was suggested as a mechanism whereby such neural changes may result in the development of psychosis [28].

A study focusing on fentanyl-induced behavioural effects utilised a zebrafish model. Animals of the same strain and background were reared, and eggs collected to assess embryo toxicity. The researcher exposed the eggs to fentanyl up to 120 h post-fertilisation. Additionally, a short-term exposure test was also conducted where fentanyl exposure to zebrafish larvae was limited to 24 h, along with a locomotor behaviour assessment. The zebrafish embryo toxicity test was used to determine the LC_50_ and EC_50_ dose for the drug. The researchers showed that fentanyl was lethal at concentrations of 67.8–102 μM with EC_50_ calculated at 10.8–24.2 μM. The sublethal toxicity effects included a slower heart rate, pericardial oedema, and yolk sac oedema, along with chorda and mouth abnormalities post-hatching. Short-term exposure (24 h) of fentanyl at lower concentrations showed no lethal or sublethal effects on zebrafish larvae. However, fentanyl did significantly reduce the locomotor activity of zebrafish larvae at and above 1 μM, demonstrating compromised behaviour from fentanyl exposure even at lower doses [29].

Similarly, fentanyl-induced neurobehavioral toxicity was investigated in adult wild-type zebrafish (6 to 8 months old). In this study, the zebrafish were treated with an intraperitoneal injection on days 3 and 5 of fentanyl at differing doses of 10, 100, or 1000 mg/L. The data showed that the higher doses fentanyl induced place preference via an increased number of zebrafish being present in the drug-paired zone and reduced cohesion in shoaling behaviour in groups of zebrafish. In individual zebrafish, fentanyl at all tested concentrations altered neurobehavioral profiles as measured via reduced anxiety-related behaviour, reduced exploratory behaviour, increased aggressive behaviour, reduced social preferences, and evidence of impaired cognitive learning and memory capacity. The neurobehavioral toxicity evidenced was explained by the disruption of neurotransmitters in the brain of zebrafish [30]. 

Elsewhere, a 2023 investigation was undertaken to study the toxicity-induced behavioural effects of two fentanyl analogues (ocfentanil and 2-furanylfentanyl). In this study, two experimental models were utilised (AB wild type zebrafish and adult male ICR CD-1 mice). Zebrafish larvae were exposed to the analogues at doses of 1 μM and 10 μM, while mice were exposed to a single dose at a concentration range of 0.1–15 mg/kg. The data showed that both fentanyl analogues significantly impaired locomotor activity in zebrafish larvae, evidenced via the reduced distances travelled by larvae. This impairment in activity was more apparent following exposure to 2-furanylfentanyl, especially at the highest tested concentration. Similarly, locomotion activity in mice was also impaired by both fentanyl analogues. 2-furanylfentanyl and ocfentanil at the lowest dose increased locomotor activity in the mice; however, both analogues administered at higher doses of 6 or 15 mg/kg significantly reduced and/or inhibited locomotor activity in mice. Finally, the authors showed that the fentanyl analogue 2-furanylfentanyl at 10 μM also induced morphological effects in zebrafish larvae, including yolk sac oedema [31].

### 3.4. Respiratory Toxicity and Depression 

Moreover, Sprague–Dawley rats received a single dose of fentanyl intravenously at a dose of 40 or 80 μg/kg. The study also used an in vitro model of Caco-2 (colon-derived epithelial cell line) in the presence of fentanyl alone and fentanyl in combination with a P-glycoprotein (P-gp) inhibitor. The data showed that fentanyl significantly increased the ATPase activity of the P-gp membrane and affected the efflux ratio of Caco-2 cells, which was then reduced when in the presence of both fentanyl and a P-gp inhibitor, showing fentanyl to be a P-gp substrate. The authors suggest that fentanyl can affect P-gp, which, in turn, is important in the drug’s ability to bypass the blood–brain barrier. It was also found that fentanyl at 80 µg/kg in combination with a P-gp inhibitor resulted in severe respiratory toxicity, determined using a whole-body plethsmograph device which simultaneously measured the fentanyl-induced effects on minute ventilation, breaths per minute, expiration time, and inspiration time. Changes to such parameters were denoted as fentanyl-induced respiratory depression/toxicity [32].

Respiratory depression is a commonly encountered, yet potentially fatal, effect following fentanyl exposure. Hill et al. (2020) studied fentanyl-induced respiratory depression in vivo, utilising male and female mice of two phenotypes. These phenotypes were μ-opioid receptor knockout mice (a strain of mice that lack the genes that encode for the μ-opioid receptor) and wild-type C57BL/J mice. In this study, the depression of respiration was measured in freely moving mice following intravenous fentanyl administration. The data showed that fentanyl at a dose of 112 μg/kg significantly depressed the minute volume with the fastest onset rate of depression compared to the other tested opioids. Moreover, fentanyl induced a decrease in tidal volume and in respiration rate. The intraperitoneal administration of fentanyl at concentrations of 0.05 to 1.35 mg/kg resulted in the depression of the minute volume, tidal volume, and respiratory rate in the wild-type strain mice. Fentanyl at the same concentrations administered to the μ-opioid receptor knockout mice, however, produced no depressant effect on these respiratory factors [33].

In 2022, an in situ arterial perfusion was performed (male and female Sprague–Dawley rats) before intubation with fentanyl at an apneic concentration to measure respiratory output in relation to the dorsolateral pontine neuronal activity. Fentanyl at a concentration of 300 nM reduced respiratory output by slowing the three-phase output pattern present at baseline conditions, transitioning this respiratory output to a two-phase pattern which too was slowed down due to the fentanyl-induced longer inspiratory and expiratory duration, resulting in sustained apnea. In a fraction of the preparations, the respiratory output following fentanyl exposure shifted from baseline to apnea directly without the presence of a slowed two-phase pattern. Fentanyl-induced apnea occurred in all tested preparations and was explained via fentanyl-induced alterations in inspiratory and expiratory neuron activity. Fentanyl at 300 nM reduced the inspiratory neuron firing frequency and, in the majority of preparation, completely silenced the inspiratory neuron activity, whereas most expiratory neurons continued to actively fire following fentanyl exposure, only affected by a reduced firing rate. Combined, these data suggest that fentanyl-induced changes in respiration ultimately result from this reduced activity in the dorsolateral pontine inspiratory neurons [34].

It is suspected that the muscle rigidity induced by fentanyl might also affect the respiratory mechanics and metabolism. To investigate this, male Sprague–Dawley rats were intravenously administered 150 or 300 μg/kg of fentanyl. Fentanyl at both concentrations reduced inspiratory activity. In 56% of rats, this decreased compliance was abrupt (90 s after fentanyl administration) and induced rhythmic contractions of the skeletal muscles, which were followed by tonic/tetanic contractions of these same muscles. This decreased respiratory compliance dropped from 0.51 ± 0.11 mL/cmH_2_O to 0.26 ± 0.06 mL/cmH_2_O, suggesting that fentanyl caused a decrease in the elasticity of the respiratory system. Decreased respiratory compliance in the other tested rats was gradual but present, reaching the reported lower level of respiratory compliance after 30 min of fentanyl exposure. Fentanyl-induced muscle contractions also increased oxygen consumption in the rat, leading to a metabolism-induced hypoxemia, an effect contributing to fentanyl-induced toxicity, along with the changes in respiratory compliance caused by fentanyl [35].

In 2019, muscle rigidity with regard to respiration was investigated with carfentanil, a highly potent fentanyl analogue. In this study, male Sprague–Dawley rats were exposed to the drug subcutaneously at doses of 1, 3 or 10 μg/kg. The authors showed that carfentanil decreased body temperature in the rats in a dose-dependent manner (carfentanil at 3 μg/kg induced a significant hypothermic response that lasted up to 4 h; at 10 μg/kg, it caused significant hypothermia in the rat that was endured up to 8 h following initial administration). Catalepsy (characterised by the rigidity of muscles) was also induced by carfentanil in a dose-dependent manner. Carfentanil-induced catalepsy showed the chest and abdominal muscles to be distressed, commonly referred to as ‘wooden chest syndrome’, which was accompanied by the rats’ laboured breathing, evidencing the induced respiratory depression caused by the drug. This study also indicated that the impaired clearance of carfentanil quantified as higher plasma concentrations of carfentanil and norcarfentanil (a carfentanil metabolite) [36].

### 3.5. In Vitro Studies

In a 2021 study, a study on the mechanisms of toxicity of a range of psychoactive substances, including fentanyl as a reference compound, was undertaken. Toxicity was investigated in vitro using dopaminergic-differentiated SH-SY5Y cells. The 15–1000 μM treatment for 2 h resulted in a concentration-dependent decrease in cell viability. Fentanyl concentrations of 125 and 250 μM were used to investigate the mechanisms of cell death through the generation of reactive oxygen species (ROS) production and subsisting apoptotic and necrotic cell death [37].

In another study, fentanyl-induced neurotoxicity was assessed in neuron cells (CRL 10742) at concentrations of up to 10 μg/mL. Initially, this study investigated the inflammation by assessing changes in alterations in the level of tumour necrosis factor-α (TNF-α), IL8, and IL10. It was noted that the fentanyl-induced increased IL8 and TNF-α gene expression was accompanied by a downregulation of IL10. Furthermore, the authors showed that fentanyl exposure inhibited paraoxonase (PON1) activity in the neuron cells (an antioxidant role) [38].

A study by Xiao et al. (2022) investigated the effect of fentanyl exposure on ovarian cancer cells in combination with chemotherapy drugs (cisplatin or paclitaxel). The study was conducted using cells in the exponential growth phase from three human ovarian cancer cell lines (SK-OV-3, TOV-21G, and SW626 cells) exposed to the drugs at a concentration range for 12 h to 3 days. Additionally, and concurrently, severe combined immunodeficiency (SCID) mice were treated daily with a fentanyl injection around the tumour site at concentrations of 10, 20, and 40 ng/kg. Ovarian cancer cell migration, proliferation, and survival were considered with respect to fentanyl’s effect. The analysis showed that fentanyl dose-dependently significantly increased cell migration by up to 2.3–3.5 times in all tested ovarian cancer cell lines, significantly increased ovarian cancer growth by up to 1.9–2.4 times, and stimulated cell proliferation to a greater extent in ovarian cancer cells compared to normal cells at concentration of 100–400 nM. Similar results were found in the animal model where fentanyl at 40 ng/kg significantly enhanced tumour growth by 1.5 times. Critically, the conclusions in this study showed that fentanyl administered in combination with cisplatin or paclitaxel significantly inhibited apoptosis compared to the chemotherapy drugs administered alone. The authors suggest that fentanyl disrupts the pro-apoptotic effects of chemotherapy drugs, thereby hindering their effectiveness in the treatment of ovarian cancer. Lastly, this study showed that fentanyl specifically activated EGFR, important in ovarian cancer [39].

Similarly, elsewhere, human umbilical vein endothelial cells (HUVECs), isolated from the human umbilical cord vein, were used to assess fentanyl toxicity via angiogenesis assays. These assays showed that the administration of fentanyl at 0.1, 1, and 10 μM increased HUVEC tube formation (10 μM stimulated tube formation by 2.3 times). These data show fentanyl acting as a pro-angiogenic opioid (Feng et al., 2021). Moreover, the effect of fentanyl on the growth, migration, and survival of HUVEC was also investigated. Fentanyl increased cell proliferation at concentrations of 0.1 to 10 μM; however, it caused cell death at higher concentrations. The authors also show that fentanyl decreased HUVEC apoptosis, as evidenced by the mechanical decrease in pro-apoptotic Bax protein without any reported effect on the Bcl-2 level. These activities of fentanyl on endothelial cells were determined to be μ-opioid receptor-independent via evidence of failure to reverse such effects. These findings taken together, this study indicates that fentanyl can display pro-angiogenic, pro-proliferative, and anti-apoptotic properties [40].

### 3.6. Rare Fentanyl-Induced Effects

Along with the common expected effects in opioid users, other less common toxic effects following fentanyl exposure have been reported. In one such case, a rare fentanyl-induced effect, cardiomyopathy, was described in a 35-year-old male following recreational use of non-prescribed fentanyl via oral consumption [41]. The initial examination of the patient found signs of consistent tachycardia with no murmurs present, tachypnoea, and hypotension. The toxicology test showed the presence of opioids - fentanyl was identified by a metabolite score of 292 (less than 10 ng/mL). A point-of-care ultrasound (POCUS) showed that the patient had signs of cardiogenic shock via a severely low ejection fraction (percentage of blood ejected of only 15–20%) indicating dysfunction in the left ventricle, resulting from fentanyl-induced cardiomyopathy. Additionally, the patient also suffered induced respiratory failure from cardiogenic pulmonary oedema. This patient had no medical history of cardiac problems prior to this case. In summary, this case suggests that fentanyl can induce cardiomyopathy and acute onset heart failure, characterised by the reduced ejection fraction [41].

Elsewhere, opioid-associated amnestic syndrome (OAS) has been a reported effect caused by fentanyl use. In a recent case, a 31-year-old male with a history of drug use was admitted for unexplained amnesia that lasted longer than 24 h following relapse, following intravenous fentanyl abuse. An initial examination showed that the patient was alert, yet could not spontaneously recall objects presented to him 5 min prior. A toxicology test showed the presence of fentanyl and norfentanyl (a fentanyl metabolite) at concentrations of 0.2 ng/mL and 23.3 ng/mL with no other substances present. The use of a head CT and MRI imaging showed hypoattenuation along the bilateral hippocampi and high signal activity with restricted diffusion, demonstrating induced bilateral hippocampi injury. All other tests performed were negative; yet, memory function was not significantly regained. Given the absence of other variables, this case was diagnosed as a case of OAS which appeared to be a persistent condition. This case presents how the use of fentanyl can result in persistent amnesia in an individual and cause injury to the bilateral hippocampi; the mechanism of injury is unknown [42].

A case of opioid-induced movement disorder has been reported at a hospital in Japan where the patient suffered complications after undergoing a surgery with the use of general endotracheal anaesthesia that included fentanyl [43]. The patient was a 22-year-old male with no reported history of drug abuse. The patient demonstrated signs of increasing agitation and pain post-operation despite the administration of additional fentanyl doses totalling 250 µg. Following further anaesthesia, the patient experienced episodes of involuntary muscle contractions throughout the entire body that occurred every 5 min, and each episode persisted for 20–40 s along with associated tachycardia, eventually leading to desaturation in the patient, with the requirement for ventilation. The patient also experienced signs of hypotension, bradycardia, and hallucinations. Following the cessation of opioid administration, the muscle contraction episodes stopped; yet, when opioid administration resumed, the muscle contraction episodes resurfaced. Considering the clinical observations, this case was concluded to be an example of opioid-induced movement disorder by which the inclusion of fentanyl, and other opioids, in the post-operative regime can induce persistent episodes of whole-body muscle contractions [43].

Unusually, fentanyl-induced effects have also been reported in paediatric cases (irregular since opioids are not prescribed to the young demographic) [44,45]. In one such case, a 9-month-old female was found to be cyanotic, unresponsive, and in critical need of ventilation following the accidental ingestion of fentanyl. Upon examination, the patient showed signs of severe bradypnea via a respiratory rate of only 6 breaths per minute as well as an elevated heart rate and blood pressure levels, along with physical notations of lethargy, involuntary jerking movements, and miotic pupils, which is a common indicator of drug ingestion. Urine tests confirmed the presence of fentanyl and further examinations concluded there to be no underlying abnormalities that may have contributed to the effects experienced by the patient [44].

Elsewhere, a 3-year-old girl was found mentally altered following the exploratory ingestion of unknown quantity opiates. Upon fossilisation, CT and MRI imaging showed the bilateral hypoattenuation of the cerebellar hemispheres with restricted diffusion—these findings are consistent with an opioid-induced malignant cerebellar oedema, along with signs of early hydrocephalus. The patient required sedation for ventilation purposes and therefore received further fentanyl dose of 0.5 µg/kg on day 0 and 1 µg/kg on day 1, which was later converted into a continuous fentanyl infusion of 2 µg/kg/h. Following sedation using fentanyl at a cumulative total of 229 µg, the patient was noted to have become autonomically unstable due to hypertension and bradycardia. Further imaging showed the persistence of the cerebellar oedema, with a resulting herniation and worsened hydrocephalus that required draining. Fentanyl administration was discontinued in the patient to avoid further complications yet her follow up examinations showed motor impairments [45].

**Figure 1:**
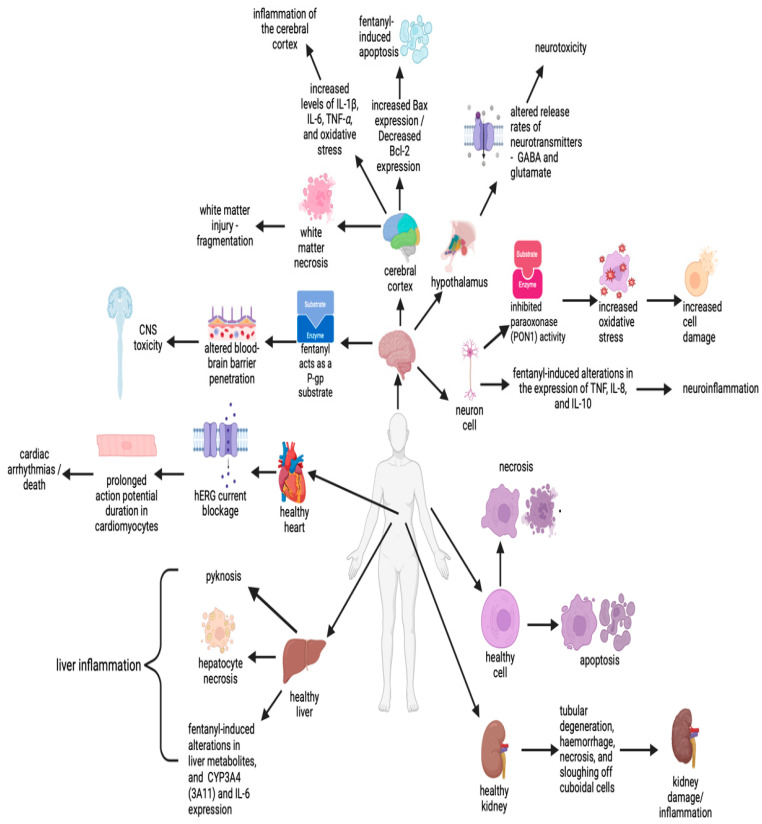
Summation of fentanyl-induced adverse effects to human health.

The mechanism of fentanyl toxicity from experimental settings is summarised in Figure 1.

### 3.7. Drug–Drug Interactions

Another study considered drug–drug interactions by which the documented effects were induced following the use of the fentanyl patch in conjunction with cocaine in a 37-year-old female [46]. This case reported the patient enduring drug-induced seizures, with the initial examination demonstrating signs of autonomic instability including tachycardia, hypertension, hyperthermia, tachypnoea, and reduced respiratory rate. The patient also suffered drug-induced behavioural effects which were presented as agitation to the point of restraint and drug-induced muscle rigidity of the full body, effects commonly reported following fentanyl toxicity. These drug-induced effects in conjunction resulted in the clinical diagnosis of serotonin syndrome, a condition that associates the presented symptoms with an excess of serotonin present in the central and peripheral nervous system, resulting in the intubation of the patient for the management of this condition [46].

### 3.8. Post-Mortem Case Studies Involving Fentanyl and/or Fentanyl Analogues

Interestingly, over the last decade, more frequent post-mortem cases have been reported that have attributed the patient’s death to fentanyl and/or its analogues. In one such study, conducted in Jefferson Parish, LA, USA, using post-mortem data from deaths between 2013–2018, 197 deaths were reported as fentanyl-related. In 165 of these cases, the drug was present in the deceased. Of the analogues detected among these fentanyl-related deaths during the 6-year period, the average concentration of fentanyl was determined at 17.62 ng/mL, acetyl fentanyl at 103 ng/mL, furanyl fentanyl at 5.93 ng/mL and acryl fentanyl at 1.42 ng/mL. The fentanyl analogues cyclopropyl fentanyl and methoxyacetyl fentanyl were also detected post-mortem but not quantified for these analogues [47].

On a similar theme, the Camden Opioid Research Initiative analysed post-mortem data from opioid-related toxicity deaths that occurred in South New Jersey, USA (2019–2021). Post-mortem blood samples collected 0–2 days after death were used for the analysis of 42 cases of opioid-related deaths. From this, the data showed that fentanyl was present in 41 of the 42 opioid-related deaths (fentanyl and norfentanyl). The average concentration of fentanyl detected was 16 ng/mL, while the average concentration of norfentanyl was 2 ng/mL [48].

In a 2019 study, fentanyl and/or its analogues were quantified in 48 post-mortem samples using ELISA. The presence of fentanyl was confirmed in 14 of these cases (0.5–230 ng/mL), with acryl fentanyl confirmed in 11 cases (0.02–12 ng/mL), cyclopropyl fentanyl in 14 cases (3.4–36 ng/mL), tetrahydrofuranyl-fentanyl in 13 cases (2–26 ng/mL), and 4-fluoroisobutyrfentanyl in 10 patients (0.76–370 ng/mL) [49].

Another study investigated the post-mortem fentanyl concentrations present in bone and bone marrow from opioid-related death cases [42]. This study involved 22 cases in Belgium that were confirmed to be opioid-related via the presence of selected opioids, one of which being fentanyl (2018–2020). In this set of trials, the clavicle bone from each deceased individual was removed and utilised. Of the 22 opioid-related cases, 4 of them were determined to be related to fentanyl. Further analysis of these 4 cases showed a fentanyl presence in skeletal tissue and blood. Fentanyl was present in the post-mortem blood samples at concentrations ranging from 1.2 to 9.2 ng/mL, while the fentanyl concentration in bone and bone marrow was 1.4–3.6 ng/g and 1.1–25 ng/g, respectively [50].

A 2019 study was conducted using post-mortem samples collected in Detroit, MI, USA between 2015 and 2017, used to quantify acetyl fentanyl. During this time, there was a total of 104 deaths relating to the use of acetyl fentanyl. All these cases had been determined as overdose deaths. The concentration of acetyl fentanyl in the serum was 0.9–37.4 ng/mL [51].

In an interesting 2020 investigation, cyclopropyl fentanyl was quantified post-mortem in a 24-year-old male who presented at autopsy with findings of liver and kidney congestion and pericardial sac. The blood, urine, and vitreous humour were collected for toxicological testing. In this experiment, liquid chromatography–tandem mass spectrometry was used to quantify the cyclopropyl fentanyl present in serum that contributed to the resulting death of the individual—20.4 ng/mL [52].

Cyclopropyl fentanyl was also quantified post-mortem in another fatal case study occurring in Zurich, Switzerland [45]. In this study, a 39-year-old male presented at autopsy with findings of brain oedema, lung congestion, and fluid in parts of the respiratory system. Blood, whole blood, urine, and gastric content samples were collected 11 or 29 h post-mortem for toxicological analysis. The data showed the presence of cyclopropyl fentanyl in femoral blood at a concentration of 19.8 ng/mL, urine at 6.6 ng/mL, and gastric content at 720 ng/mL [53].

Moreover, in a UK study conducted between February 2017 and January 2018 from 84 deaths with a suspicion of fentanyl poisoning and a history of heroin use, the presence of fentanyl and/or a fentanyl analogue was detected in 40 of the individuals post-mortem. The fentanyls detected were carfentanil, fentanyl, 4-fluorobutyrylfentanyl, or butyrylfentanyl [54]. 

Finally, in a 2018 retrospective study conducted in Lithuania, the analysis of 63 post-mortem patients whose suspected cause of death was drug overdose showed the presence of carfentanyl in the blood and urine of 15 patients [55].

## 4. Discussion and Conclusions

The incidence of heroin-related overdose deaths has stabilised in recent years, whereas deaths attributed to other synthetic opioids have continued to increase. Novel synthetic opioids represent an emerging group of novel psychoactive substances which include fentanyl-related compounds. Additionally, and importantly, in recent years, due to its potency and low cost, drug dealers have been mixing fentanyl with other drugs including heroin, methamphetamine, and cocaine, increasing the likelihood of a fatal interaction. This has contributed to an increase in drug-related overdose deaths. New analogues of fentanyl from illegal sources are continually evolving, making the detection of relevant metabolites in patients difficult. Importantly, some of the newer psychoactive substances or new/new synthetic analogues are not yet regulated or even tested. Therefore, the full adverse effects on human health for these new products may not yet be fully understood.

From the literature, it is clear that the fentanyl is highly cytotoxic, with necrotic and apoptotic cell death identified in experimental settings [37]. It is apparent that the drug causes a range of adverse effects in the human body, affecting a range of systems including the liver, with inflammation and changes in normal behaviour (i.e., [23]), neurobehavioral toxicity via a disruption of normal neurotransmitter activity (i.e., [30]), and respiratory depression via an alteration in the activity of inspiratory neurons (i.e., [34]). It is understood that 2 mg of fentanyl can be lethal to the average human, although previous usage will have an influence on this [56]. The variety of organs affected, combined with the different mechanism of toxicity for fentanyl (described above), is highly concerning, as it cannot be assumed that fentanyl will affect individuals similarly and might manifest in a variety of unpredictable adverse outcomes. These idiosyncratic outcomes have no doubt contributed to the ever-increasing numbers of fentanyl-related deaths in recent years.

In addition to fentanyl, there is a growing body of data reporting the toxicity-induced effects of numerous fentanyl analogues, including ocfentanil, 2-furanylfentanyl, and carfentanil. It seems that there is some similarity between the effects reported for fentanyl and its analogues (i.e., [31,35,36]). Importantly, however, the potency of the analogues differs considerably. Obviously, this is extremely important with regard to the adverse effects of the drugs on human health and subsequent fatal cases. It is very clear that further research is required to study these analogues and their toxicity profiles.

In addition to traditional in vitro (cell lines and primary cells) and in vivo models (predominantly rodents), the zebrafish model has gained popularity in assessing fentanyl-induced adverse effects. The literature suggests that there are some similarities in fentanyl toxicity between zebra fish and the traditional rodent models. As an example, fentanyl was shown to have induced a reduced heart rate in the zebrafish model [29], indicating signs of fentanyl-induced respiratory depression in this model, which is on par with the rodent model whereby fentanyl was shown to have induced respiratory depression in wild-type C57BL/J strain mice [33] (although the doses and route of exposure of the drug used between these studies are very different). However, from the literature, it is abundantly clear that further research is required to validate the zebrafish model as a suitable complementary model for testing drug toxicity.

The post-mortem toxicological data reported for fentanyl and its analogues could prove useful as indicators of the doses that might result in fatality. However, as described in detail above, extreme differences between post-mortem concentrations for fentanyl and its analogues are apparent in the literature. However, the post-mortem studies suggest that the demographic mostly responsible for fentanyl/analogues abuse are white males aged 20–40 years old (i.e., 47–53). This demographic information is useful to recognise as it means that this population deemed mostly affected by fentanyl fatalities could somewhat be targeted when raising awareness of the dangers imposed by fentanyl abuse in the affected demographic.

This review provides a comprehensive insight into the toxicity of fentanyl and its various analogues. The scrutiny of post-mortem cases aids in our current understanding of the relationship between the drugs and fatality and the demographic currently abusing fentanyl.

## Data Availability

Not applicable.
